# Making External Validation Valid for Molecular Classifier Development

**DOI:** 10.1200/PO.21.00103

**Published:** 2021-08-05

**Authors:** Yilin Wu, Huei-Chung Huang, Li-Xuan Qin

**Affiliations:** ^1^ Department of Epidemiology and Biostatistics, Memorial Sloan Kettering Cancer Center, New York, NY

## Abstract

**PURPOSE:**

Accurate assessment of a molecular classifier that guides patient care is of paramount importance in precision oncology. Recent years have seen an increasing use of external validation for such assessment. However, little is known about how it is affected by ubiquitous unwanted variations in test data because of disparate experimental handling and by the use of data normalization for alleviating such variations.

**METHODS:**

In this paper, we studied these issues using two microarray data sets for the same set of tumor samples and additional data simulated by resampling under various levels of signal-to-noise ratio and different designs for array-to-sample allocation.

**RESULTS:**

We showed that (1) unwanted variations can lead to biased classifier assessment and (2) data normalization mitigates the bias to varying extents depending on the specific method used. In particular, frozen normalization methods for test data outperform their conventional forms in terms of both reducing the bias in accuracy estimation and increasing robustness to handling effects. We make available our benchmarking tool as an R package on GitHub for performing such evaluation on additional methods for normalization and classification.

**CONCLUSION:**

Our findings thus highlight the importance of proper test-data normalization for valid assessment by external validation and call for caution on the choice of normalization method for molecular classifier development.

## INTRODUCTION

Precision medicine needs effective quantitative tools for outcome prediction to tailor treatment choices and optimize patient care.^1,2^ Molecular profiling technologies herald the promise for developing such tools.^3–6^ However, few of the published molecular classifiers have been successfully translated into clinical use so far.^7–10^ Often a classifier was reported to be effective based on cross-validation in the initial publication, but later failed external validation in an independent test data set.^11–14^

**CONTEXT**

**Key Objective**
External validation is increasingly used for assessing the accuracy of a molecular classifier, yet little is known about test-data normalization for removing ubiquitous unwanted variations because of disparate experimental handling.
**Knowledge Generated**
We showed that data normalization mitigates the negative impact of unwanted variations to varying extents depending on the specific method used. In particular, frozen normalization methods for test data outperform their conventional forms.
**Relevance**
Our findings highlight the importance of proper test-data normalization for valid assessment by external validation and call for caution on the choice of normalization method for molecular classifier development.

We recently showed that some of these failures can be attributed to biased classifier assessment by cross-validation when training data possess handling effects (namely systematic data variations because of disparate experimental handling of the specimens) and subsequently undergo data normalization. The reason is that normalization can lead to overcompressed data variability and hence overoptimistic assessment of the classification error rate.^15,16^ It remains to be elucidated how these failed classifiers were influenced by handling effects and their normalization for test data in external validation.

Although external validation is increasingly used in recent studies of molecular classification, many of these studies failed to report the normalization method used for test data.^17–22^ Among those that did report, a jumble of methods was used, including median normalization (MN) and quantile normalization (QN), either applied to test data alone or in combination with training data.^23–25^ To date, there has been no systematic study on the relative performance of the normalization methods for test data.

In this paper, we studied the issues of test data handling effects and normalization in the context of microRNA (miRNA) microarrays.^6,26,27^ Our study used two data sets for the same set of tumor samples, which were previously collected at Memorial Sloan Kettering Cancer Center (MSK).^28–30^ Arrays in one data set were collected with uniform handling to minimize handling effects and balanced array-to-sample allocation to avoid any confounding, whereas arrays in the other data set were collected with nonuniform handling and unbalanced allocation. We then performed resampling-based simulations using the paired data sets, dubbed virtual rehybridization, to create additional data under various levels of handling effects and biologic signals and different designs for allocating arrays to samples. In this paper, we report our findings from this simulation study. These findings provide critical insights in developing reproducible miRNA classifiers for clinical application.

## METHODS

### Empirical Data Collection

A set of 192 untreated primary gynecologic tumor samples (96 endometrioid endometrial tumors and 96 serous ovarian tumors) were collected at MSK from 2000 to 2012. Human tumor tissues of the 192 samples were obtained from participants who provided informed consent and their use in our study was approved by the MSK Institutional Review Board. The samples were profiled using the Agilent Human miRNA Microarray (Release 16.0; Agilent Technologies, Santa Clara, CA), following the manufacturer's protocol. This array platform contains 3,523 markers (representing 1,205 human and 142 human viral miRNAs) and multiple replicates for each marker (ranging from 10 to 40). In addition, it has eight arrays on each glass slide (ie, an experimental block) arranged as two rows and four columns. Two data sets were obtained from the same set of samples using different methods of experimental handling. The first data set (hereafter referred to as the uniformly handled data set) was handled by one technician in one batch with the arrays assigned to tumor samples using blocked random assignment. By contrast, the second data set (hereafter referred to as the nonuniformly handled data set) was handled by two technicians over *multiple batches* in the order of sample collection; the first 80 arrays were handled by one technician in *two batches* and the last 112 by a second technician in *three batches*. More details on data collection can be found in the studies by Qin et al.^28,30^

### Resampling-Based Simulation

As a proof of concept, we used tumor type (endometrial cancer *v* ovarian cancer) as the outcome variable for classification. The specific steps of the resampling-based simulation are as follows.

1. First, we used the uniformly handled data set to approximate the biologic effects for each sample. Among a total of 3,523 markers on the array, 351 (10%) were significantly differentially expressed (*P* < .01) between the two tumor types. To be consistent with the typical signal strength in a molecular classification study,^31–33^ we halved the between-group differences of biologic effects for the 351 significant markers (by deducting a half of the ovarian-versus-endometrial between-group differences from their levels of expression in ovarian samples), reducing the number of significant markers to 63 (2%). The resulting biologic effects served as virtual samples. They were split *randomly* in a 2:1 ratio into a training set (n = 128) and a test set (n = 64), balanced by tumor type.
2. Second, we used the difference between the two arrays (one from the uniformly handled data set and the other from the nonuniformly handled data set, subtracting the former from the latter) for the same samples to approximate the handling effects for each array in the nonuniformly handled data set. These handling effects served as virtual arrays. They were split *nonrandomly* to a training set (n = 128, the first 64 arrays and the last 64 arrays) and a test set (n = 64, the middle 64 arrays). By definition, handling effects are systematic effects that are not reproducible in different data sets; therefore, virtual arrays are split *nonrandomly* so that they are not comparable between training data and test data. The magnitude of handling effects in training data and test data was then adjusted by adding a constant to training data and multiplying by a factor for test data. We used three settings for the constant and the multiplication factor: (1) 2 and 2, (2) 1 and 1.5, and (3) 0.5 and 1.25, mimicking the scenarios when handling effects in test data were (1) highly, (2) moderately, and (3) slightly different from those in training data, respectively.
3. Third, training data were simulated through virtual rehybridization by assigning virtual arrays to virtual samples following a *partial confounding* design or a *stratification* design, and then summing the biologic effects for a sample and the handling effects for its assigned array. A partially confounding design assigned 90% of the first 64 arrays and 10% of the last 64 arrays to *ovarian* samples and the rest of the arrays to *endometrial* samples. A stratification design assigned arrays in each batch (ie, each of the experimental batches in the collection of the nonuniformly handled data set) to the two tumor groups in equal proportion.
4. Finally, test data were simulated also through virtual rehybridization using a *partial confounding* design or a *stratification* design similar to training data. Note that, here, the partial confounding design assigned 90% of the first 32 arrays and 10% of the last 32 arrays were assigned to *endometrial* samples and the rest of the arrays to *ovarian* samples. As a *reference*, we also examined test data that comprised only biologic effects (without adding any handling effects).

One hundred simulation runs were generated for each scenario of handling-effect pattern and array-allocation design.

### Preprocessing and Analysis of the Simulated Data

The analysis for each simulated training data set followed three main steps: (1) preprocessing training data and test data; (2) building a classifier using the preprocessed training data; and (3) assessing the error rate of the classifier using the preprocessed test data. Further details are provided as below.

#### Data preprocessing.

Preprocessing of both training data and test data consisted of three steps: (1) log2 transformation, (2) across-sample normalization, and (3) marker-replicate summarization using the median.^34^ Training data were normalized with QN as the primary approach and with MN as an alternative approach. Test data were normalized by one of the six methods: (1) no normalization (NN), (2) MN, (3) QN, (4) frozen MN (fMN), (5) frozen QN (fQN; ie, mapping the empirical distribution of each individual test-set sample to the frozen empirical distribution of the normalized training data), and (6) pooled QN (pQN; ie, apply QN after pooling training data and test data).^24^

#### Classifier building.

We used Prediction Analysis for Microarrays as the primary approach for classification and Least Absolute Shrinkage and Selection Operator (LASSO) as an alternative approach.^35,36^ R packages pamr and glmnet were used for applying these methods, with the tuning parameters chosen by five-fold cross-validation.

#### Classifier assessment.

Classification accuracy was measured using the misclassification error rate (ie, the proportion of samples that were misclassified). The final model of each classifier was built using the entire training data and applied to predict the group label for each sample in test data. The predicted groups were compared with their true groups for assessing the misclassification error rate.

### Performance Measure of Test-Data Normalization

We denote the error rate based on test data that possessed handling effects as Error_HE and that based on test data free of handling effects as Error_noHE. To gauge the performance of a normalization method for abating the impact of test-data handling effects, we compared Error_HE against Error_noHE. A normalization method is effective if it removes handling effects so well that (1) its Error_HE approximates the corresponding Error_noHE and (2) its Error_HE is small.

All analyses in this paper were performed using R 3.5.0.

## RESULTS

We present here the simulation results using QN for training data and Prediction Analysis for Microarrays for classifier development, under each of the three levels of signal-to-noise ratio and the four combinations of array-to-sample allocation design. Additional results using MN for training data and LASSO for classification are provided in the Data Supplement.

### Results When Handling Effects Were Highly Different Between Training Data and Test Data

**FIG 1.**
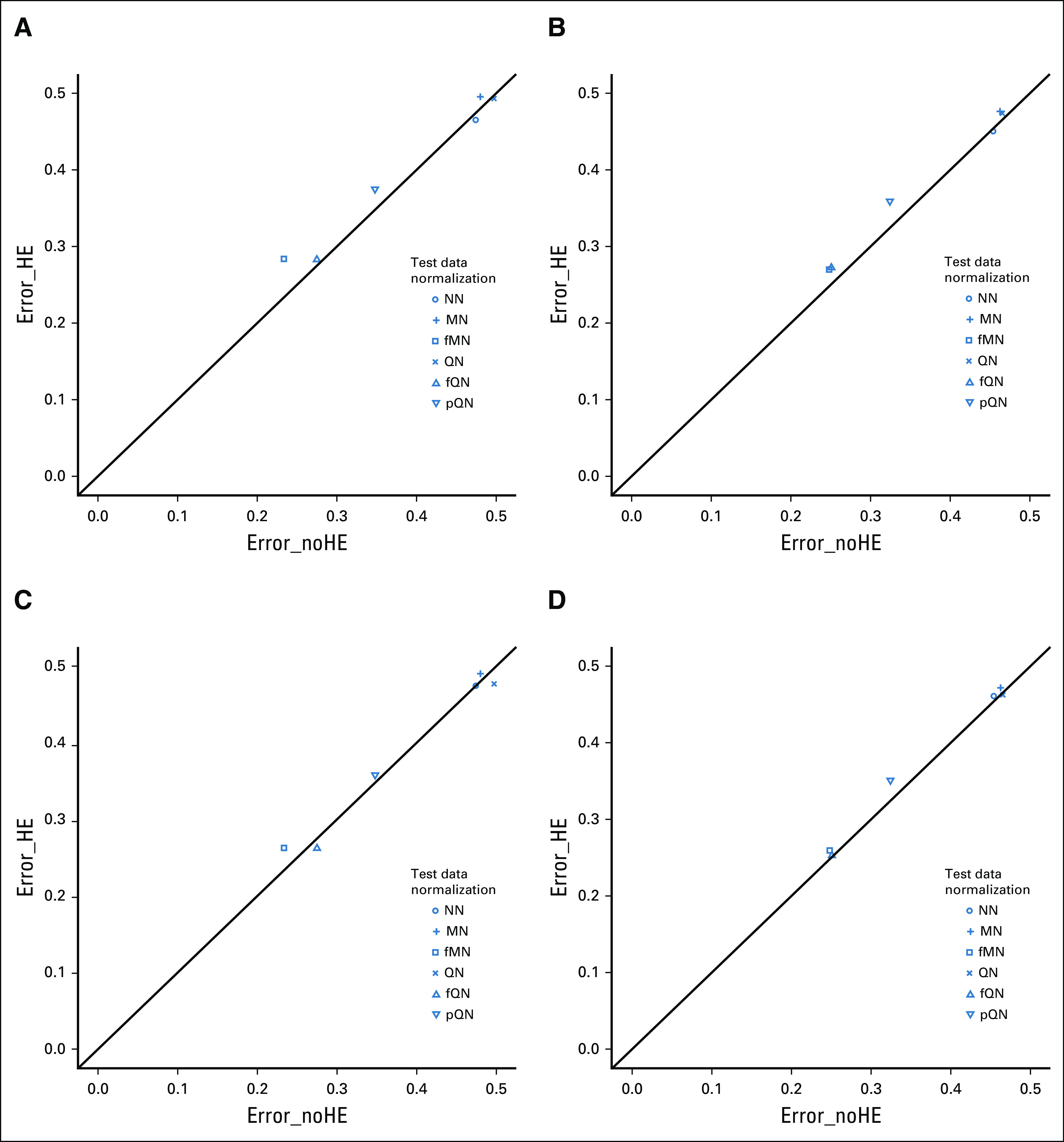
Scatterplot of the mean misclassification error rate estimated among 100 simulation runs, with the error rates based on test data free of handling effects plotted on the *x*-axis and that based on test data with handling effects on the *y*-axis. Handling effects were *highly* different between training data and test data. (A-D) Array-to-sample allocation followed a confounding design or a stratification design for training data and test data: (A) PC− training data, PC+ test data; (B) STR training data, PC+ test data; (C) PC− training data, STR test data; and (D) STR training data, STR test data. Classifiers were developed with the Prediction Analysis for Microarrays method; training data were subject to QN; test data were normalized by a method indicated by the point symbol. fMN, frozen median normalization; fQN, frozen quantile normalization; HE, handling effects; MN, median normalization; NN, no normalization; PC, partial confounding; pQN, pooled quantile normalization; QN, quantile normalization; STR, stratification.

Figure 1A shows the simulation results when handling effects in test data were highly different from those in training data. Across all four array-allocation designs, the error rate based on test data with handling effects (ie, Error_HE) ranged from 0.283 to 0.495 after normalization, compared with that without normalization, 0.465. The exact level of error rate depended on the specific normalization method used: fQN (0.283) and fMN (0.284) were the best performers, QN (0.493) and MN (0.495) were the worst, and pQN (0.375) was in the middle. These error rates were in nearly perfect agreement with these for handling-effect-free test data (ie, Error_noHE) for QN and MN, in their regular and frozen forms; the agreement for pQN was slightly worse. These observations suggested that, with this pattern of handling effects and design for array allocation, fQN and fMN were the best methods for test-data normalization as they not only effectively removed the negative impact of handling effects but also made test data more comparable to training data, leading to smaller error rates; QN and MN were the worst performers as they led to an error rate even worse than NN.

The use of stratification for training data reduced the small difference in Error_HE between fQN and fMN and that between QN and MN (Figs 1B and 1D), and its use for test data alone brought Error_HE for pQN to closer agreement with Error_noHE (Fig 1C), indicating a marginal benefit for balanced design in this simulation scenario.

### Results When Handling Effects Were Moderately Different Between Training Data and Test Data

**FIG 2.**
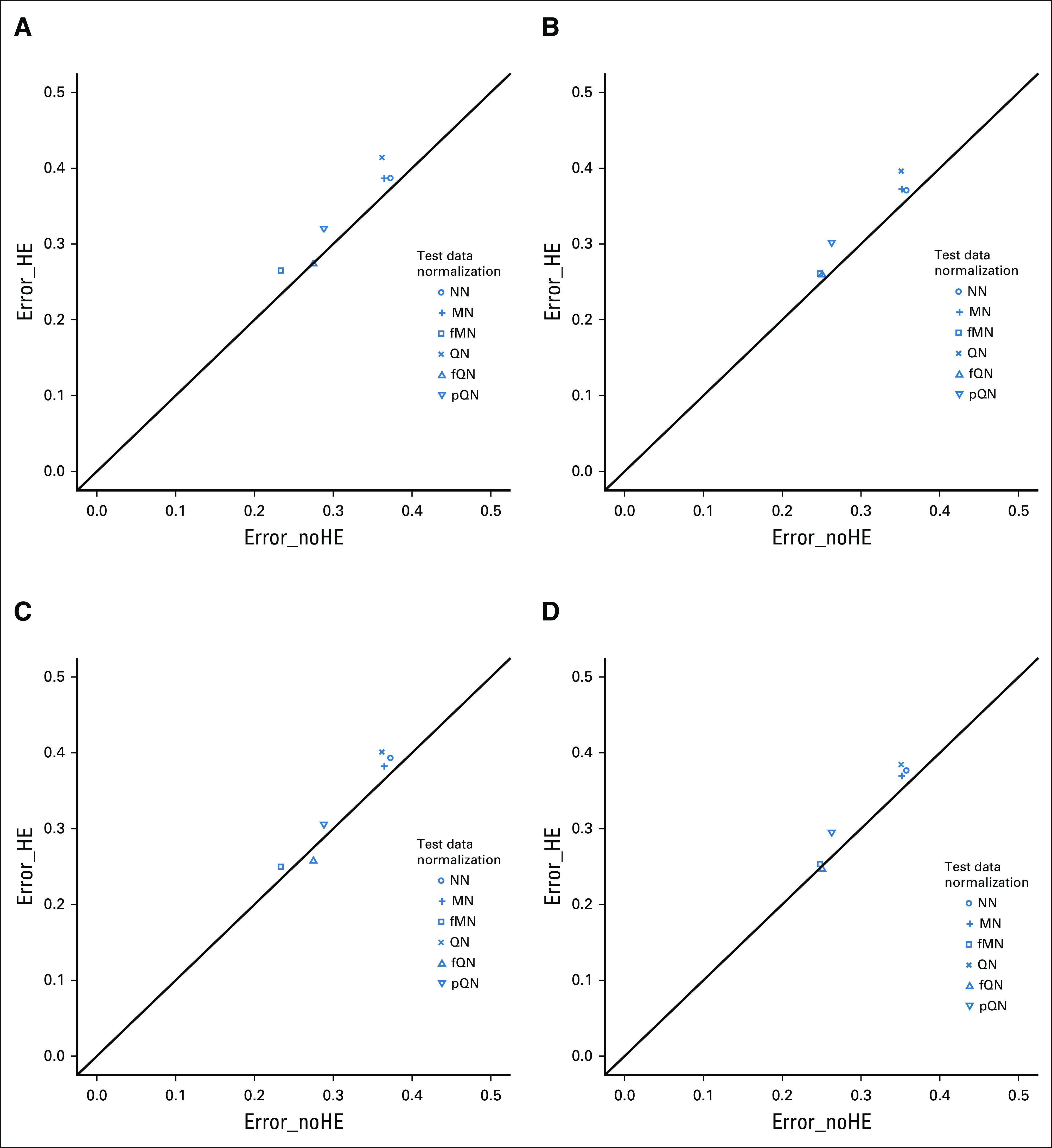
Scatterplot of the mean misclassification error rate estimated among 100 simulation runs, with the error rates based on test data free of handling effects plotted on the *x*-axis and that based on test data with handling effects on the *y*-axis. Handling effects were *moderately* different between training data and test data. (A-D) Array-to-sample allocation followed a confounding design or a stratification design: (A) PC− training data, PC+ test data; (B) STR training data, PC+ test data; (C) PC− training data, STR test data; and (D) STR training data, STR test data. Classifiers were developed with the Prediction Analysis for Microarrays method; training data were subject to QN; test data were normalized by a method indicated by the point symbol. fMN, frozen median normalization; fQN, frozen quantile normalization; HE, handling effects; MN, median normalization; NN, no normalization; PC, partial confounding; pQN, pooled quantile normalization; QN, quantile normalization; STR, stratification.

Figure 2A shows the simulation results when handling effects in test data were moderately different from those in training data. When array allocation followed the partial confounding design, Error_HE remained at a similar level for fMN (0.265) and fQN (0.274), and it decreased for pQN (0.320), MN (0.387), QN (0.414), and NN (0.387). Its level of agreement with Error_noHE was again nearly perfect for fMN and fQN and slightly worse for MN, QN, and pQN. The exact error rate also depended on the normalization method, and the relative performance of these methods stayed roughly the same, with fQN and fMN still being the best performers. The error rates associated with QN and MN decreased but their level of agreement with Error_noHE worsened slightly; the error rate for pQN also decreased while its agreement with Error_noHE remained at a similar level.

Also similar to the pervious scenario, the use of stratification design for training data again reduced the small difference in Error_HE between fQN and fMN (Figs 2B and 2D), and its use for test data alone led to better agreement with Error_noHE for pQN, QN, and MN (Fig 2C).

### Results When Handling Effects Were Slightly Different Between Training Data and Test Data

**FIG 3.**
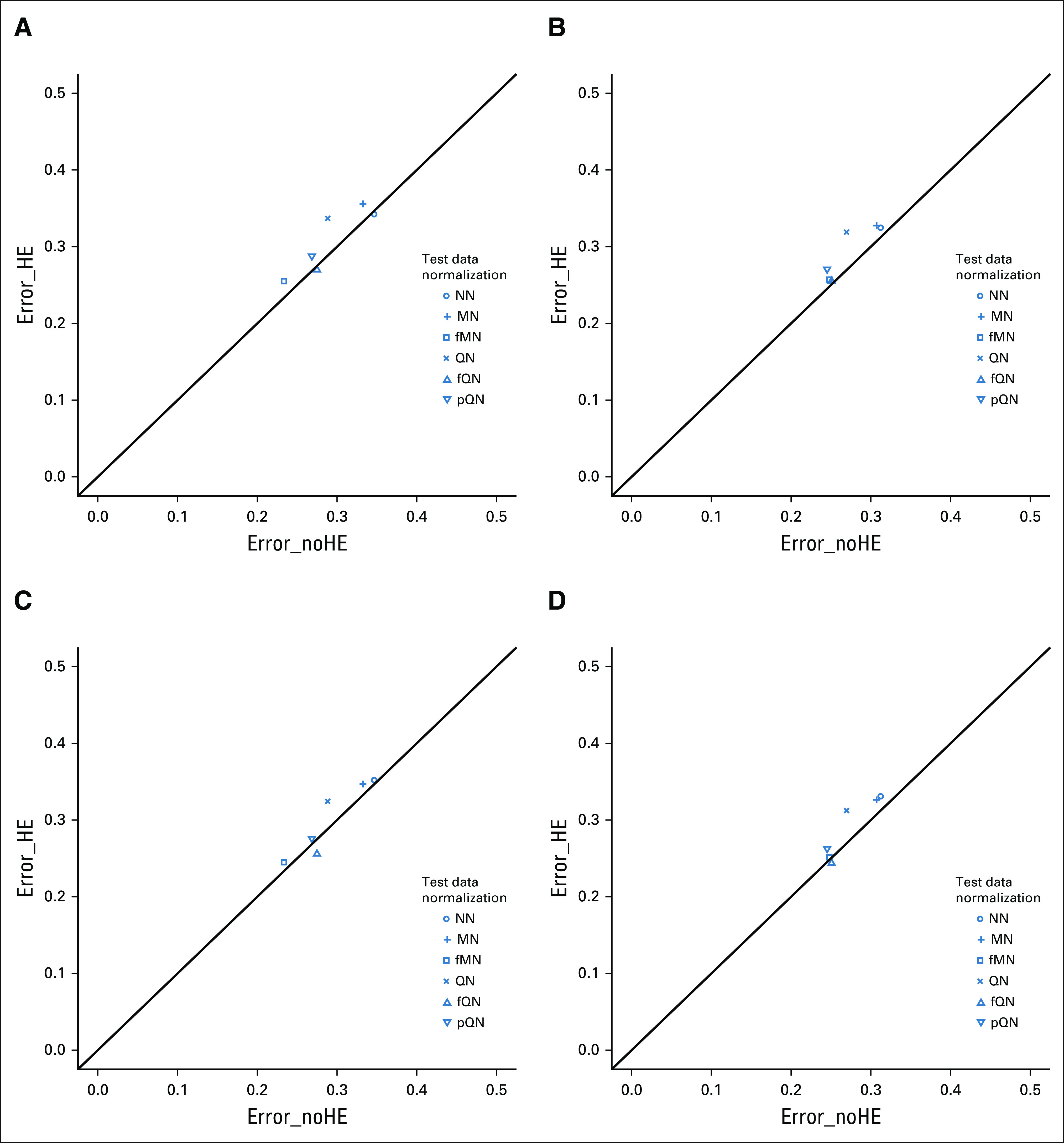
Scatterplot of the mean misclassification error rate estimated among 100 simulation runs, with the error rates based on test data free of handling effects plotted on the *x*-axis and that based on test data with handling effects on the *y*-axis. Handling effects were *slightly* different between training data and test data. (A-D) Array-to-sample allocation followed a confounding design or a stratification design: (A) PC− training data, PC+ test data; (B) STR training data, PC+ test data; (C) PC− training data, STR test data; and (D) STR training data, STR test data. Classifiers were developed with the Prediction Analysis for Microarrays method; training data were subject to QN; test data were normalized by a method indicated by the point symbol. fMN, frozen median normalization; fQN, frozen quantile normalization; HE, handling effects; MN, median normalization; NN, no normalization; PC, partial confounding; pQN, pooled quantile normalization; QN, quantile normalization; STR, stratification.

Figure 3A shows the simulation results when handling effects in test data were slightly different from those in training data. Although the relative ordering of various normalization methods remained similar, their differences became smaller when compared with the previous two scenarios. More specifically, Error_HE was 0.270, 0.255, 0.288, 0.337, and 0.356 for fQN, fMN, pQN, QN, and MN, respectively, compared with 0.342 without normalization. Their level of agreement with Error_noHE remained nearly perfect for fQN and fMN and worse for the other three methods.

The use of stratification for training data reduced the error rate for fQN (0.255, 0.244) and fMN (0.257, 0.251; Figs 3B and 3D). Its use for test data alone led to better agreement with Error_noHE for pQN (0.271) and QN (0.319; Fig 3C).

### Additional Simulation Results for Alternative Methods of Training-Data Normalization and Classification

We performed additional simulations using MN as an alternative method for training data normalization and using LASSO as an alternative method for classification. We observed similar results in terms of the relative performance of test-data normalization methods, the benefit of balanced study design, and the effect of various patterns of handling effects in training data and test data (Data Supplement).

Furthermore, we generated biologic effects parametrically using a normal distribution for each miRNA with its mean and standard deviation estimated from the empirical data. The findings remain the same qualitatively, whereas the error rates decreased across the board, possibly because of the lack of between-marker correlation when simulating the data (Data Supplement).

### Software Development for Reproducing Our Study and Examining Additional Methods

We encourage interested researchers to replicate our study and explore additional methods for data normalization and classifier development. Toward this end, we developed an R package containing the paired data sets and another R package implementing the resampling-based simulation study. These two packages, named PRECISION.array.DATA and PRECISION.array, are deposited at GitHub.^37^ The data can also be accessed at Gene Expression Omnibus via a SuperSeries record (GSE109059). The PRECISION.array package not only has implemented the methods for normalization and classification reported in this paper, but also allows additional methods specified by the user.

## DISCUSSION

In this paper, we investigated the important yet understudied problem of test-data normalization for making external validation valid. Using paired data sets and resampling-based simulations, we showed that (1) handling effects in test data can lead to biased classifier assessment and (2) test-data normalization can mitigate the bias but to varying extents depending on the method. In particular, frozen versions of QN and MN outperformed the conventional versions, especially when the pattern of handling effects is highly different between training data and test data; conventional MN and QN of test data offer limited benefits compared with NN in our simulations and can even be worse under some scenarios of handling effects.

Our findings suggest that improper choice of normalization methods for test data in published studies may have undermined validation efforts for molecular classifiers and disproved some actually useful classifiers because of improper test-data normalization. For example, using the last 64 samples in the nonuniformly handled data as test data for assessing a classifier built on the first 128 samples, the error rate was 0.391 for conventional QN but 0.297 for fQN, whereas the error rate based on the uniformly handled data of the test samples was 0.281 and 0.266, respectively. For those classifiers that were successfully validated, inadequate description of the methodology used can hamper both efforts to replicate these studies and application of the classifiers to future samples in clinical practice. For the purpose of developing accurate and reproducible molecular classifiers, we recommend using (1) uniform experimental handling in data collection to mitigate handling effects, (2) frozen normalization of quantiles or medians for test data when either training data or test data possess handling effects, and (3) comprehensive description of the study design and analysis methods in publication, ideally accompanied by software code to allow faithful replication and application.

For proof of concept, we report the simulation results for a limited number of simulation scenarios and statistical approaches for normalization and classification. We have developed two R packages, PRECISION.array.DATA and PRECISION.array, for interested researchers to use for further exploring this topic with additional simulation scenarios and statistical methods. Our simulation approach makes a working assumption that handling effects are additive to biologic effects. This assumption has been considered reasonable for microarray data and adopted in publications on microarray data normalization and analysis.^38,39^

To the best of our knowledge, the issue of data normalization for external validation has not been studied before. Our findings fill a critical knowledge gap in the advancement of developing reproducible classifiers for clinical use and speak to the importance of proper methodology and sufficient reporting.^40^

## References

[1] Pencina MJ Peterson ED Moving from clinical trials to precision medicine: The role for predictive modeling JAMA 315 1713–1714 2016 27115375 10.1001/jama.2016.4839

[2] Steyerberg EW Vergouwe Y Towards better clinical prediction models: Seven steps for development and an ABCD for validation Eur Heart J 35 1925–1931 2014 24898551 10.1093/eurheartj/ehu207 PMC4155437

[3] Hu JC, Tosoian JJ, Qi J (2018). Clinical utility of gene expression classifiers in men with newly diagnosed prostate cancer. JCO Precis Oncol.

[4] Adams JU Genetics: Big hopes for big data Nature 527 S108–S109 2015 26580158 10.1038/527S108a

[5] Dhurandhar EJ, Vazquez AI, Argyropoulos GA (2014). Even modest prediction accuracy of genomic models can have large clinical utility. Front Genet.

[6] Landi MT Zhao Y Rotunno M et al MicroRNA expression differentiates histology and predicts survival of lung cancer Clin Cancer Res 16 430–441 2010 20068076 10.1158/1078-0432.CCR-09-1736 PMC3163170

[7] Ioannidis JP Allison DB Ball CA et al Repeatability of published microarray gene expression analyses Nat Genet 41 149–155 2009 19174838 10.1038/ng.295

[8] McShane LM Altman DG Sauerbrei W Identification of clinically useful cancer prognostic factors: What are we missing? J Natl Cancer Inst 97 1023–1025 2005 16030294 10.1093/jnci/dji193

[9] Ransohoff DF Bias as a threat to the validity of cancer molecular-marker research Nat Rev Cancer 5 142–149 2005 15685197 10.1038/nrc1550

[10] Simon R Radmacher MD Dobbin K et al Pitfalls in the use of DNA microarray data for diagnostic and prognostic classification J Natl Cancer Inst 95 14–18 2003 12509396 10.1093/jnci/95.1.14

[11] McShane LM Cavenagh MM Lively TG et al Criteria for the use of omics-based predictors in clinical trials Nature 502 317–320 2013 24132288 10.1038/nature12564 PMC4180668

[12] Sebastiani P, Solovieff N, Puca A (2010). Genetic signatures of exceptional longevity in humans. Science.

[13] Akey JM Biswas S Leek JT et al On the design and analysis of gene expression studies in human populations Nat Genet 39 807–808 2007 author reply 808-809 17597765 10.1038/ng0707-807

[14] Baggerly KA Morris JS Edmonson SR et al Signal in noise: Evaluating reported reproducibility of serum proteomic tests for ovarian cancer J Natl Cancer Inst 97 307–309 2005 15713966 10.1093/jnci/dji008

[15] Rahman M Jackson LK Johnson WE et al Alternative preprocessing of RNA-Sequencing data in The Cancer Genome Atlas leads to improved analysis results Bioinformatics 31 3666–3672 2015 26209429 10.1093/bioinformatics/btv377 PMC4804769

[16] Qin LX Huang HC Begg CB Cautionary note on using cross-validation for molecular classification J Clin Oncol 34 3931–3938 2016 27601553 10.1200/JCO.2016.68.1031 PMC5477984

[17] Onken MD Winkler AE Kanchi KL et al A surprising cross-species conservation in the genomic landscape of mouse and human oral cancer identifies a transcriptional signature predicting metastatic disease Clin Cancer Res 20 2873–2884 2014 24668645 10.1158/1078-0432.CCR-14-0205 PMC4096804

[18] Ferté C Trister AD Huang E et al Impact of bioinformatic procedures in the development and translation of high-throughput molecular classifiers in oncology Clin Cancer Res 19 4315–4325 2013 23780890 10.1158/1078-0432.CCR-12-3937 PMC3745509

[19] Watanabe T Kobunai T Yamamoto Y et al Prediction of liver metastasis after colorectal cancer using reverse transcription-polymerase chain reaction analysis of 10 genes Eur J Cancer 46 2119–2126 2010 20570135 10.1016/j.ejca.2010.04.019

[20] Wang Y Klijn JG Zhang Y et al Gene-expression profiles to predict distant metastasis of lymph-node-negative primary breast cancer Lancet 365 671–679 2005 15721472 10.1016/S0140-6736(05)17947-1

[21] Lee JS Leem SH Lee SY et al Expression signature of E2F1 and its associated genes predict superficial to invasive progression of bladder tumors J Clin Oncol 28 2660–2667 2010 20421545 10.1200/JCO.2009.25.0977

[22] Brueffer C, Vallon-Christersson J, Grabau D (2018). Clinical value of RNA sequencing-based classifiers for prediction of the five conventional breast cancer biomarkers: A report from the population-based multicenter Sweden cancerome analysis network-breast initiative. JCO Precis Oncol.

[23] Schwalbe EC Lindsey JC Nakjang S et al Novel molecular subgroups for clinical classification and outcome prediction in childhood medulloblastoma: A cohort study Lancet Oncol 18 958–971 2017 28545823 10.1016/S1470-2045(17)30243-7 PMC5489698

[24] McCall MN Bolstad BM Irizarry RA Frozen robust multiarray analysis (fRMA) Biostatistics 11 242–253 2010 20097884 10.1093/biostatistics/kxp059 PMC2830579

[25] Korkola JE Houldsworth J Feldman DR et al Identification and validation of a gene expression signature that predicts outcome in adult men with germ cell tumors J Clin Oncol 27 5240–5247 2009 19770384 10.1200/JCO.2008.20.0386 PMC3651602

[26] Nair VS Maeda LS Ioannidis JP Clinical outcome prediction by microRNAs in human cancer: A systematic review J Natl Cancer Inst 104 528–540 2012 22395642 10.1093/jnci/djs027 PMC3317879

[27] Shih KK Qin LX Tanner EJ et al A microRNA survival signature (MiSS) for advanced ovarian cancer Gynecol Oncol 121 444–450 2011 21354599 10.1016/j.ygyno.2011.01.025

[28] Qin LX, Huang HC, Villafania L (2018). A pair of datasets for microRNA expression profiling to examine the use of careful study design for assigning arrays to samples. Sci Data.

[29] Qin LX, Levine DA (2016). Study design and data analysis considerations for the discovery of prognostic molecular biomarkers: A case study of progression free survival in advanced serous ovarian cancer. BMC Med Genomics.

[30] Qin LX Zhou Q Bogomolniy F et al Blocking and randomization to improve molecular biomarker discovery Clin Cancer Res 20 3371–3378 2014 24788100 10.1158/1078-0432.CCR-13-3155 PMC4079727

[31] van't Veer LJ Dai H van de Vijver MJ et al Gene expression profiling predicts clinical outcome of breast cancer Nature 415 530–536 2002 11823860 10.1038/415530a

[32] Girard L Rodriguez-Canales J Behrens C et al An expression signature as an aid to the histologic classification of non-small cell lung cancer Clin Cancer Res 22 4880–4889 2016 27354471 10.1158/1078-0432.CCR-15-2900 PMC5492382

[33] Li Y, Kang K, Krahn JM (2017). A comprehensive genomic pan-cancer classification using The Cancer Genome Atlas gene expression data. BMC Genomics.

[34] Qin LX Huang HC Zhou Q Preprocessing steps for agilent microRNA arrays: Does the order matter? Cancer Inform 13 105–109 2014 10.4137/CIN.S21630 PMC4560483 26380547

[35] Tibshirani R The lasso method for variable selection in the Cox model Stat Med 16 385–395 1997 9044528 10.1002/(sici)1097-0258(19970228)16:4<385::aid-sim380>3.0.co;2-3

[36] Tibshirani R Hastie T Narasimhan B et al Diagnosis of multiple cancer types by shrunken centroids of gene expression Proc Natl Acad Sci USA 99 6567–6572 2002 12011421 10.1073/pnas.082099299 PMC124443

[37] Qin LX: GitHub repository. https://github.com/LXQin.

[38] Qin LX, Satagopan JM (2009). Normalization method for transcriptional studies of heterogeneous samples—Simultaneous array normalization and identification of equivalent expression. Stat Appl Genet Mol Biol.

[39] Kerr MK Martin M Churchill GA Analysis of variance for gene expression microarray data J Comput Biol 7 819–837 2000 11382364 10.1089/10665270050514954

[40] Altman DG Sauerbrei W McShane LM Importance of the distinction between quality of methodology and quality of reporting HPB (Oxford) 19 649–650 2017 28438426 10.1016/j.hpb.2017.02.444

